# The mechanism and performance of rock breakage by undercutting disc cutter with advanced slotting

**DOI:** 10.1038/s41598-023-37576-1

**Published:** 2023-06-28

**Authors:** Hongxiang Jiang, Xiaodi Zhang, Huihe Zhao, Zenghui Liu, Yongxin Wang

**Affiliations:** 1 School of Mechatronic Engineering, China University of Mining and Technology, Jiangsu Province, Xuzhou, 221116 People’s Republic of China; 2 Jiangsu Province and Education Ministry Co-Sponsored Collaborative Innovation Center of Intelligent Mining Equipment, Xuzhou, 221008 People’s Republic of China; 3 College of Mechanical and Electrical Engineering, Hohai University, Nanjing, 210024 People’s Republic of China

**Keywords:** Mechanical engineering, Civil engineering

## Abstract

To study the mechanism and performance of rock breakage by an undercutting disc cutter with advanced slotting, a three-dimensional numerical model of rock cutting by a disc cutter with advanced slotting assistance was established based on the discrete element method. The parallel bond constitutive model was selected to describe the micromechanical properties of rock. The correctness of the established numerical model is verified through rock breakage experiments, and the rock cutting process by the disc cutter was analyzed by a combination of the force chain and crack distribution. The influencing factors, such as advanced slotting depth, cutting thickness, rock strength, and cutter rotation speed, on rock cutting performance were investigated. The results show that a compact zone is gradually formed between the rock and disc cutter at the beginning, then a large number of microscopic tensile and shear cracks in the compact zone due to micro failure of rock are formed; the subsequent main rock fragment is mainly caused by tensile failure; advanced slotting can reduce the rock bearing capacity and bending resistance, the rock above the advanced slotting fractured easily due to its lower bending resistance, and the volume compact zone is relatively small. When the advanced slotting depth is equal to 12.5 mm, the propulsive force and specific energy consumption of rock cutting by the disc cutter are reduced by 61.6% and 16.5%, respectively. The propulsive force and specific energy consumption increase as the rock strength increases, but they tend to close when the rock strength is greater than 80 MPa, which indicates that advanced slotting assistance is more suitable for hard rock. The results obtained in this paper can provide the operating parameters determination under different factors to some extent of the undercutting disc cutter in a pre-cut condition, which further improve the rock breaking performance of mechanized cutter.

## Introduction

Restricted by factors such as geological conditions, underground working space and rock excavating technology, the average excavation speed of underground rock roadways is less than 80 m/month, which means that the roadway excavation speed cannot satisfy the needs of underground coal mining^1^. Thus, there is an urgent need to develop a mechanized continuous cutting method for underground hard rock (uniaxial compressive strength ≥ 100 MPa). At present, the mechanized excavation of rock roadways is mainly based on point pick and disc cutter: point pick is difficult to cut the hard rock efficiently, and the pick wear and failure is continually; TBM (Tunnelling boring machine) disc cutter using extrusion and shearing manner can cut hard rock due to its huge pulling force, but the TBM is very difficult to be used in underground medium-short distance rock roadways due to its large volume and turning radius; the undercutting disc cutter is a great potential manner for underground hard rock excavation due to its high rock breakage ability and low cutting force, but it also exist cutter wear and broken problem^2^. Many scholars have investigated hard rock breakage by disc cutter to improve the hard rock breakage capacity and efficiency.

Tan et al.^3^ employed the DEM to study the rock breakage process and concluded that the propulsive force is related to internal crack propagation. Considering that the fracture characteristic and crack propagation of the rock is affected by many factors, scholars conducted the research of effect of various factors, including the structure and mounting parameters of disc cutter^4^, confining pressure and cutting sequence^5^, structure and working parameters of cutter head^6,7^, on the rock breaking performance, and abundant results are provided. Furthermore, the results reported in literature^8–10^ indicated that the disc cutter cutting in a free face condition exerts a lower cutting load and its cutting ability improves correspondingly, which further proved that the free face is conducive to rock fragmentation. The undercutting disc cutter, proposed by Ramezanzadeh and Hood^11^ by improving the structure and working mode of the TBM disc cutter, precisely makes full use of the free face of the rock to break it.

After that, scholars and engineers investigate the cutting mechanism of the undercutting disc cutter. Moxham^12^ compared the rock cutting performance of disc cutter working in the undercutting mode with the conventional rock cutting mode, and proved that the crack is more likely to propagate induced by the free face and forming the larger fragments. Jiang et al^13^ believed that the rock failure mode can change from compression or shear failure to tension failure dominance, which is conducive for crack propagation, causing lower specific energy. Moreover, the research on the effect of the structure and cutting parameters of the undercutting disc cutter on the rock breaking performance has also been carried out by the scholars. Kotwica^14^ developed a novel cutting head mounted by the asymmetrical mini disk tools based on the undercutting disc cutter, and studied the effect of the geometry structure and motion trace of the novel cutting head on rock cutting performance and cutter wear characteristics. Stopka^15^ obtained the cutting force of the undercutting disc cutter with two cutting depths of sandstone and concrete, respectively, based on the DEM.

Nevertheless, the disc cutter operating in undercutting mode still has problems of fast wear and high failure rate in the case of cutting high strength rock, especially the uniaxial compression strength of rock exceeds 100 MPa. Therefore, many assistant approaches have been proposed, among them, the high waterjet is a more concerned, more mature means of assisting mechanical cutter to break rock. With the help of water jet, advanced slotting on the intact rock mass is beneficial to the release of rock cohesion and induces crack development and propagation, and it has been applied to assist the TBM disc cutter to break rock. Meanwhile, many scholars studied the effect of various factors, including kerf depth, kerf spacing, and pre-cutting mode, on the rock breaking performance and crack propagation when the disc cutter works in a pre-cut condition^16–21^. However, in the existing cases of disc cutter breaking rock assisted by the waterjet, the nozzle and the disc cutter are separated, which leads to a greater target distance between nozzle and rock surface and the pre-slotting effect is limited.

**Figure 1:**
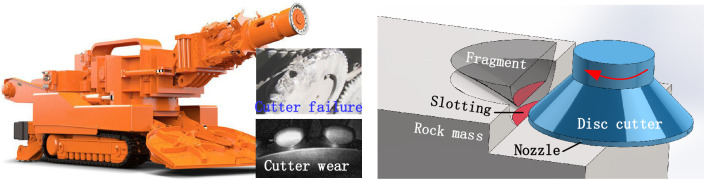
Principle of rock breaking of undercutting disc cutter with advanced slotting.

In addition, the research of advanced slotting method is mainly concentrated on the TBM disc cutter. Therefore, on the basis of the undercutting method with disc cutter, the author proposed the integration of advanced slotting technology into the disc cutter cutting rock as shown in Fig. 1^13,22^. By slotting the rocks on the cutting path of the under cutting disc cutter in advance, the difficulty of the disc cutter wedging into hard rock is reduced so that the disc cutter can make full use of the non-tensile property of the rock to wedge the hard rock and reduce the difficulty and load of the disc cutter cutting hard rock.

This paper uses a combination of numerical simulations and experiments to study the rock breakage mechanism of under cutting disc cutter with advanced slotting and analyzes the influence of factors such as advanced slotting depth, cutting thickness, rock strength on the cutting load and the specific energy consumption of rock breakage, which will provide the basis for continuous mechanized cutting of underground hard rock excavation.

## Methods

### Rock constitutive model

The particle flow numerical simulation method constructs the geotechnical material to be composed of discrete particles and simulates the geotechnical microstructure through the bonding between particles. However, the particle flow model cannot directly assign macroscopic mechanical parameters to actual geotechnical materials and indirectly matches the macroscopic mechanical parameters through mesoscopic parameter calibration. In fact, the mesoscopic parameters to be calibrated are related to the selection of the rock constitutive model, so it is necessary to select an appropriate rock constitutive model before parameter calibration.

**Figure 2:**
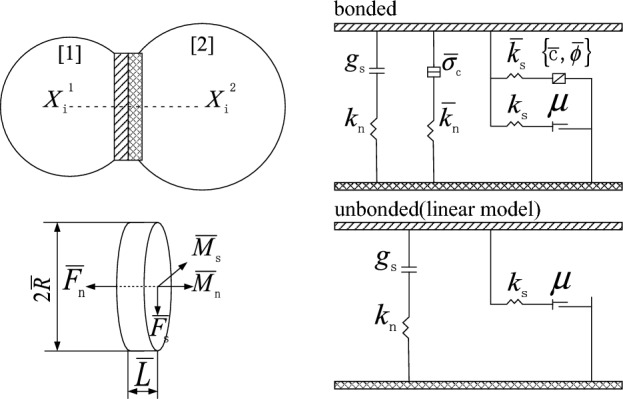
The parallel bond constitutive model.

The particle flow numerical simulation software PFC provides various constitutive models, such as the linear contact model, contact bond model, and parallel bond model. The particle contact of the parallel bond model occurs in the linear elastic bonding interface with a certain size, which can transmit forces and moments. When the stress on the interface exceeds the tensile and shear strengths given by the model, the bonding bond fails and fractures, which can better simulate the failure of internally cemented geotechnical materials. This paper uses the parallel bond model to establish a three-dimensional numerical model of the rock, and the parallel bond constitutive model is shown in Fig. 2.

The parallel bonding bond can be thought of as a set of springs with constant stiffness that act in parallel with the component springs of the linear model. When the parallel bonding bond is formed, there is no force $\overline{F}$ or torque $\overline{M}$ on the bond. When the bonded particles move relative to each other, the force $\overline{F}$ and moment $\overline{M}$ increase, which can be decomposed into the normal force $\overline{F}_{n}$, shear force $\overline{F}_{s}$, bending moment $\overline{M}_{s}$, and torque $\overline{M}_{n}$, as shown in Fig. 2. The incremental updating formula of each component force and component moment is as follows:

1 $$\begin{aligned} \overline{F}_{n} \leftarrow \overline{F}_{n} + \Delta \overline{F}_{n} = & \overline{F}_{n} + \overline{k}_{n} \overline{A}\Delta \delta_{n} \\ \overline{F}_{s} \leftarrow \overline{F}_{s} + \Delta \overline{F}_{s} = & \overline{F}_{s} + \overline{k}_{s} \overline{A}\Delta \delta_{s} \\ \overline{M}_{n} \leftarrow \overline{M}_{n} + \Delta \overline{M}_{n} = & \overline{M}_{n} + \overline{k}_{s} \overline{J}\Delta \theta_{n} \\ \overline{M}_{s} \leftarrow \overline{M}_{s} + \Delta \overline{M}_{s} = & \overline{M}_{s} + \overline{k}_{n} \overline{I}\Delta \theta_{s} \\ \end{aligned}$$

where $\Delta \overline{F}_{n}$, $\Delta \overline{F}_{s}$, $\Delta \overline{M}_{n}$ and $\Delta \overline{M}_{s}$ are the increments of normal force $\overline{F}_{n}$, shear force $\overline{F}_{s}$, torque $\overline{M}_{n}$, and bending moment $\overline{M}_{s}$, respectively; $\Delta \delta_{n}$ is the relative normal displacement increment; $\Delta \delta_{s}$ is the relative tangential displacement increment; $\Delta \theta_{n}$ is the relative torsional increment; and $\Delta \theta_{s}$ is the relative bending rotation increment.

For the three-dimensional model, the parallel bond normal stiffness $\Delta \overline{k}_{n}$, the parallel bond tangential stiffness $\Delta \overline{k}_{s}$, the cross-sectional area $\overline{A}$, the moment of inertia of the parallel bond cross-section $\overline{I}$, and the polar moment of inertia of the parallel bond cross-section $\overline{J}$ in Eq. (1) can be obtained from the following formulas:

2 $$\overline{k}_{n} = \overline{E}^{*} /L,\overline{k}_{s} = \overline{k}_{n} /\overline{k}^{*}$$

3 $$\overline{A} = \pi \overline{R}^{2} ,\;\overline{I} = \frac{1}{4}\pi \overline{R}^{4} ,\;\overline{J} = \frac{1}{2}\pi \overline{R}^{4}$$

4 $$\overline{R} = \overline{\lambda }\min \left( {R^{\left( 1 \right)} ,\;R^{\left( 2 \right)} } \right)$$

5 $$L = R^{\left( 1 \right)} + R^{\left( 2 \right)}$$

where $\overline{E}^{*}$ is the parallel bond elastic modulus, $\overline{k}^{*}$ is the parallel bond stiffness ratio, $\overline{\lambda }$ is the radius multiplier, and $R^{(1)}$ and $R^{(2)}$ are the radii of the bonded particles.

Based on elastic beam theory, the maximum tensile stress and shear stress of the parallel bond between particles are obtained:

6 $$\begin{aligned} \overline{\sigma } = & \frac{{\overline{F}_{n} }}{{\overline{A}}} + \frac{{\left\| {\overline{M}_{s} } \right\|\overline{R}}}{{\overline{I}}} \\ \overline{\tau } = & \frac{{\left\| {\overline{F}_{s} } \right\|}}{{\overline{A}}} + \frac{{\left\| {\overline{M}_{n} } \right\|\overline{R}}}{{\overline{J}}} \\ \end{aligned}$$

**Figure 3:**
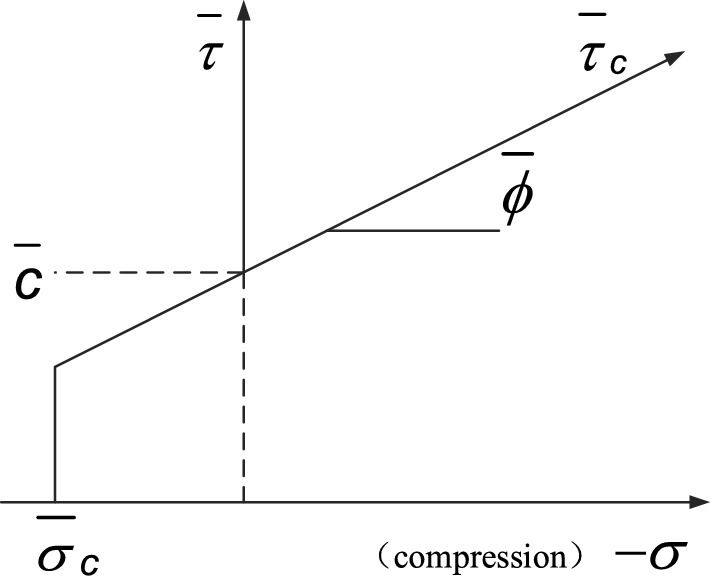
Failure envelope of the parallel bond model.

The Fig. 3 shows the failure envelope of the parallel bond model, where $\overline{\sigma }_{c}$ is the parallel bond tensile strength and $\overline{\tau }_{c}$ is the shear strength given by the model. From the tangential behavior of the parallel bonding model in Fig. 2, $\overline{\tau }_{c}$ is related to the cohesion $\overline{c}$ and the friction angle $\overline{\varphi }$, $\overline{\tau }_{c} = c - \sigma \tan \overline{\varphi }$, where $\sigma = \overline{F}_{n} /\overline{A}$. When the tensile stress or shear stress of the parallel bonding bond exceeds the given strength limit, the parallel bonding bond fails and fractures. At this time, the bond, force, moment and stiffness are deleted from the numerical model, and the parallel bond model degenerates into a linear model, as shown in Fig. 3.

### Parameter calibration

At present, there is no efficient and fast method to calibrate the mesoscopic parameters of rock, and a time-consuming "trial-and-error method" is still used; that is, by constantly adjusting the mesoscopic parameters, the results of uniaxial compression numerical tests are matched with the macroscopic mechanical parameters of rock‒soil. However, the "trial and error method" itself has high uncertainty, and the large number of meso-parameters to be calibrated increases the difficulty of parameter calibration, so it is necessary to simplify the meso-parameters. According to the research results of POTYONDY^23^, taking *R*_*m*_/*R*_*n*_ = 1.66, the rock particle stiffness ratio $k^{*} = \overline{k}^{*}$, and the rock particle elastic modulus $E^{*} = \overline{E}^{*}$. The "trial and error method" was used to calibrate the mesoscopic parameters of the medium-strength sandstone, and the macroscopic mechanical parameters of the sandstone uniaxial compressive test are shown in Table 1.

**Table 1:** Macroscopic mechanical parameters of sandstone.

Parameters	Easticity modulus ${\text{E}}_{\text{r}}$/GPa	Poisson's ratio ${\text{v}}_{\text{r}}$	Compressive strength $\overline{\sigma }_{r}$/MPa
Test	22.97	0.22	52.52
Simulation	22.57	0.21	51.35
Error	1.74%	3.18%	2.23%

Based on the parallel bond model, a standard rock sample with a size of *ϕ*50 × 100 mm was established, the minimum particle radius *R*_*n*_ was 0.9 mm, the particle density was selected as the sandstone density of 2580 kg/m^3^, and a set of initial mesoscopic parameters was set. According to the research of Zhou^24^, when the number of particles on the minimum scale of the model is RES ≥ 10, the particle size and number have little effect on the macroscopic parameters of the model. The calculation formula of RES is:

7 $$RES = \left( {L_{{\text{a}}} /R_{n} } \right)\left[ {1 + R_{m} /R_{n} } \right]$$

where *L*_*a*_ is the minimum scale of the model.

**Figure 4:**
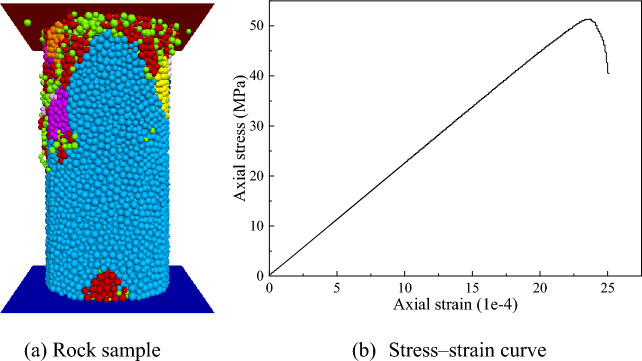
Uniaxial compression simulation test of sandstone.

In the numerical model of the sandstone uniaxial compression simulation, RES is approximately equal to 20.9, which satisfies the condition. The uniaxial compression numerical simulation was carried out by assigning the loading speed to the upper and lower walls, as shown in Fig. 4. Based on the research results of Deng et al.^25–27^, parameter calibrations are carried out in the order of the parallel bond stiffness ratio $\overline{k}^{*}$, parallel bond effective modulus $\overline{E}^{*}$, parallel bond tensile strength $\overline{\sigma }_{c}$ and parallel bond cohesion $\overline{c}$. The calibration is considered complete when the error of the macroscopic mechanical parameters of the numerical simulation and test is within ± 5%. The calibrated sandstone microscopic parameters are shown in Table 2.

**Table 2:** Microscopic parameters of simulated sandstone.

${\overline{E} }^{*}$/GPa	${\overline{k} }^{*}$	${\tilde{\sigma }}_{\mathrm{c}}$/MPa	$\overline{c }$/MPa	$\overline{\lambda }$
16.0	1.6	18.0	30.0	1.0

**Figure 5:**
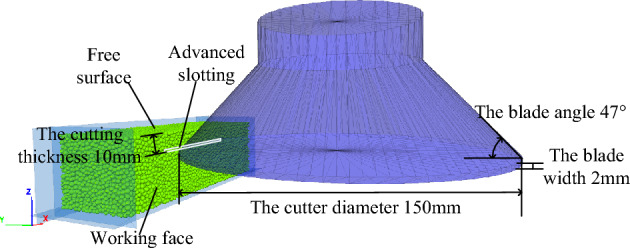
The three-dimensional model of rock breaking with a disc cutter assisted by advanced slotting.

Based on the calibrated meso-parameters, a rock numerical model with a size of 120 × 40 × 35 mm was established. The model consists of 24,555 particles whose mechanical properties are shown in Table 3, the top and right walls are deleted and defined as free surfaces, the remaining four walls are retained to limit the rock model, and the particles in contact with the four walls are grouped and completely fixed. According to the actual size of the disc cutter, the cutter model is established in the SolidWorks software, and the model is imported through the wall import command to generate an infinite-stiffness wall to simulate the disc cutter. The wall attribute command is used to apply displacement to the cutter wall to move it to a specified position. Advanced slotting is realized by deleting the rock particles on the cutting path of the disc cutter in real time. The three-dimensional model of rock breaking with a disc cutter assisted by advanced slotting is shown in Fig. 5.

**Table 3:** The mechanical properties of particles in DEM.

Minimum radius/mm	*R*_*max*_*/R*_*min*_	Porosity	Mean coordination number	Friction coefficient
1.25	1.66	0.36	3.5	0.45

### Numerical model verification

**Figure 6:**
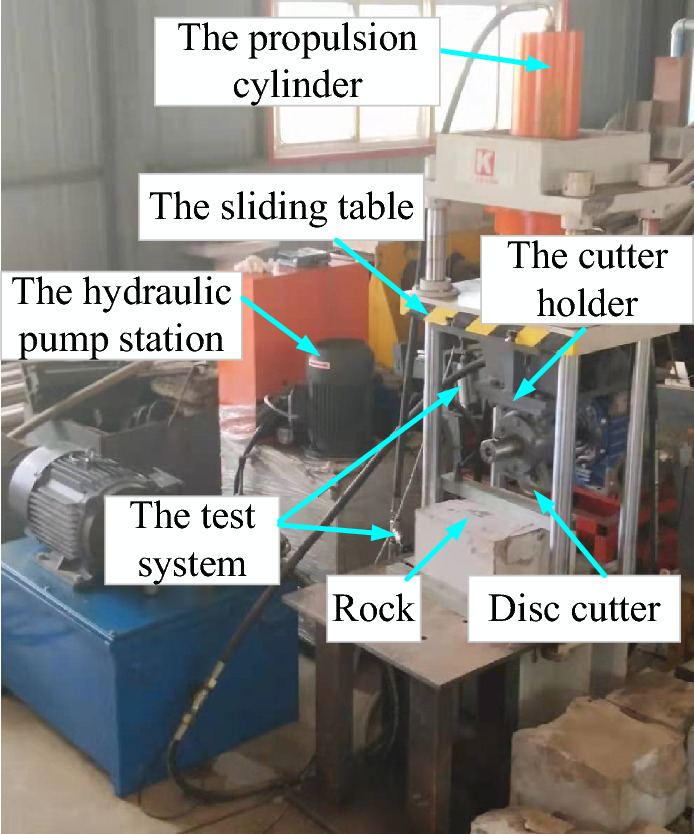
Test device for rock breaking by disc cutter extruding.

To verify whether the established numerical model can correctly simulate the rock-breaking process of the cutter, a rock-breaking experiment and numerical simulation of cutter extrusion were carried out. Figure 6 shows the test device of rock breaking by disc cutter extruding, which is mainly composed of a hydraulic pump station, propulsion cylinder, sliding table, cutter holder, disc cutter, rock and test system. The sliding table and the cutter holder are moved vertically downward by pushing the cylinder so that the disc cutter extrudes the rock, and the extrusion load of the disc cutter is calculated from the measured oil cylinder pressure.

**Figure 7:**
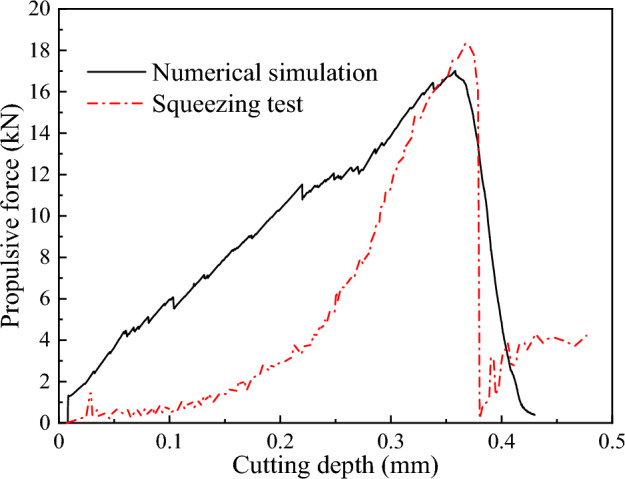
The cutter propulsive force curve of the experiment and numerical simulation.

Under the condition of a rock cutting thickness of 20 mm, the cutter propulsive force curve of the test and numerical simulation is shown in Fig. 7. The load of the disc cutter fluctuates greatly in the numerical simulation, and the contact load stiffness between the disc cutter and the rock is relatively low at the initial stage of the test load. The fluctuation of the propulsive force of the disc cutter in the numerical simulation is due to the large size of the rock particles and the short-term weak contact caused by the failure of the bonding bonds of the rock particles under the action of disc cutter extrusion. At the initial stage, the propulsive force of the test rises slowly with the increasing extrusion distance of the disc cutter, which is mainly affected by the installation clearance of the disc cutter, the material of the disc cutter, and the compression characteristics of the hydraulic oil, resulting in low contact stiffness between the disc cutter and rock at the initial loading stage. In addition, the disc cutter in the numerical model is an ideal rigid body, and its stiffness is much greater than that of the experimental cutter, which is also the reason for the difference between the propulsive force of the experiment and the numerical simulation. Figure 7 shows that the peak load of the experiment is 18,321.9 N, the peak load of the numerical simulation is 17,012.3 N, and the error is 7.1%, which verifies the effectiveness of the proposed modeling method.

**Figure 8:**
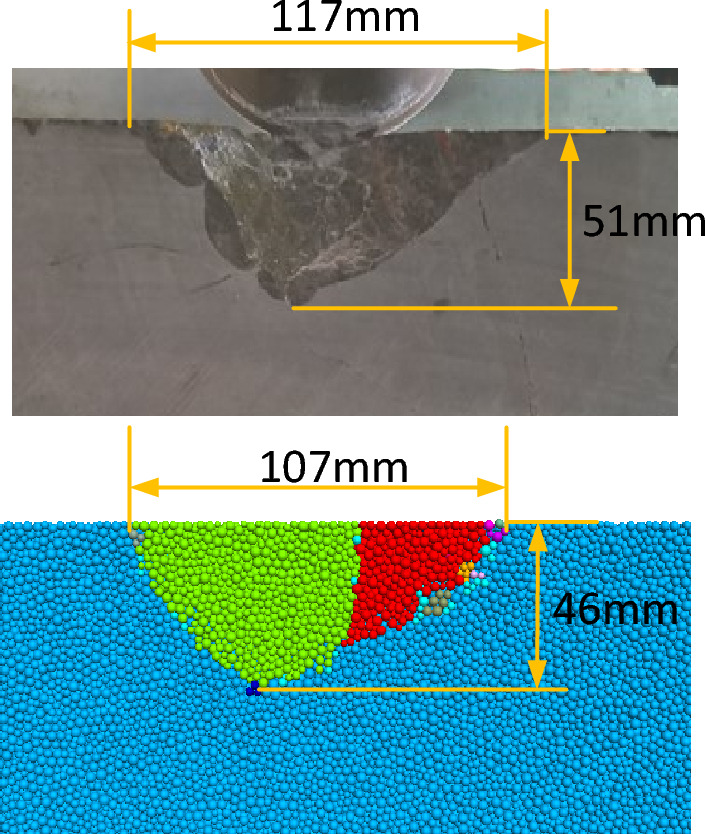
Comparison of rock fragmentation.

The rock fragmentation of the experiment and the numerical simulation is shown in Fig. 8. It can be seen that the shapes of the rock compression fractures are similar. The length of the rock fragment of the experiment and the numerical simulation is 117 mm and 107 mm, and the height is 51 mm and 46 mm, respectively. The errors in the length and height of the rock fragments are 8.5% and 9.8%, respectively.

**Figure 9:**
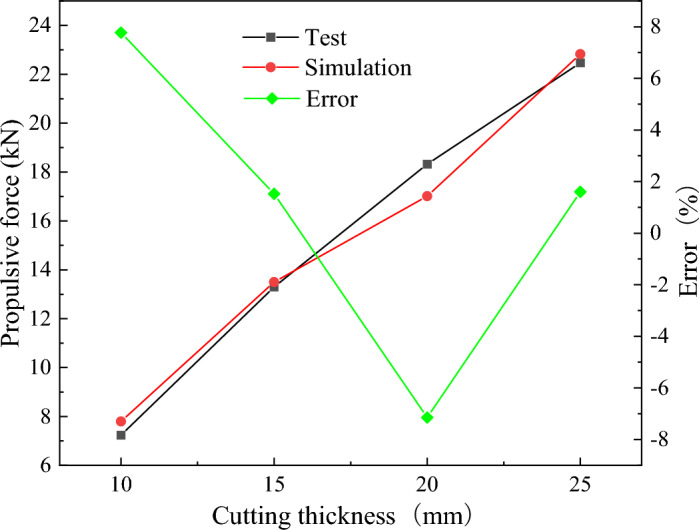
The propulsive force of the disc cutter extruding rock under different cutting thicknesses.

On this basis, the experiments and numerical simulation of disc cutter extruding rock were carried out with cutting thicknesses of 10 mm, 15 mm and 25 mm. The peak changes of the propulsive force of the test and numerical simulation under different cutting thicknesses are shown in Fig. 9. The peak propulsive force of the disc cutter extruding the rock increases with increasing cutting thickness. Due to the influence of the experimental device, operation error, and particle size, there is an error between the peak propulsive force of the numerical simulation and the experiment, but the error is less than 10%. Therefore, the numerical model method proposed in this paper can better reproduce the rock breaking process and load characteristics of cutter extrusion.

## Analysis of the rock breaking mechanism of disc cutter cutting

### Analysis of the rock breaking mechanism of disc cutter cutting

**Figure 10:**
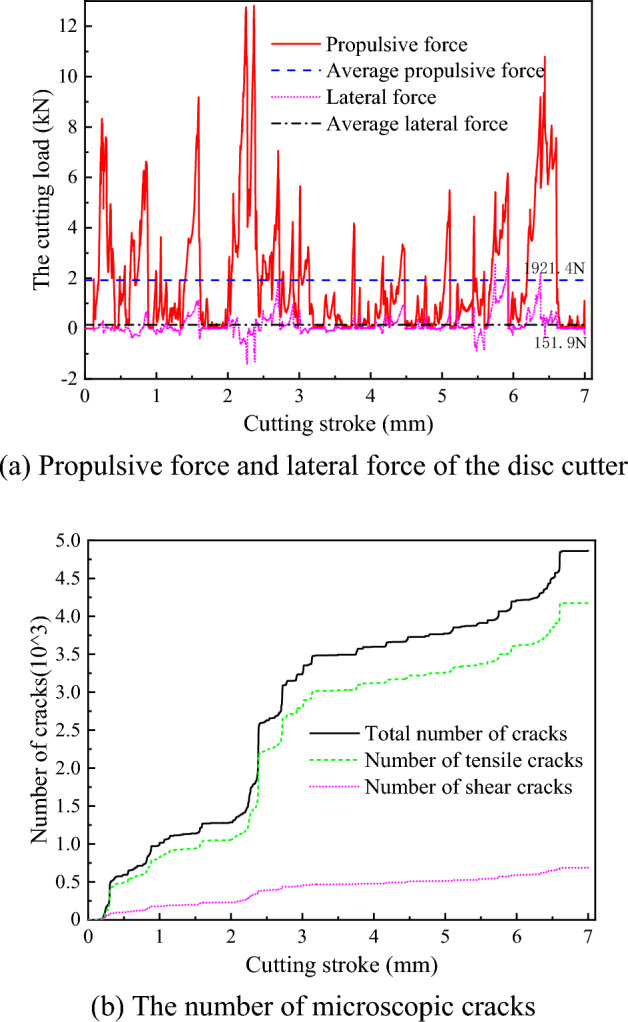
The cutting load and the number of microscopic cracks of the disc cutter cutting rock without advanced slotting.

To study the rock breaking mechanism and performance of disc cutter cutting, a discrete element model of hard rock mass (simulated bluestone) with a uniaxial compressive strength of 122 MPa was established. The rotation speed of the disc cutter is 50 rad/s, the feed rate is 0.1 m/s, and the cutting thickness is 10 mm. Figure 10 shows the cutting load and the number of microscopic cracks of the disc cutter cutting rock without advanced slotting. The disc cutter is always subjected to the positive pressure exerted by the rock in the cutting direction, and the lateral force of the cutter shows positive and negative fluctuations. The average propulsive force of 1921.4 N is much larger than the average lateral force of 151.9 N. Combined with the number in Fig. 10b, it can be seen that the propulsive force of the disc cutter gradually increases to the peak value when microscopic cracks are not initiated, and the propulsive force of the disc cutter decreases rapidly from the peak value with the generation and expansion of rock cracks.

In addition, it can be seen from Fig. 10b that the number of total cracks and tensile cracks increases in an approximately stepwise manner dramatically. The main reason for this phenomenon is due to the rock being a quasi-brittle material, and the tensile fracture plays an important role under the action of the cutter which is indicated in Fig. 10b that the number of tensile cracks is obviously greater than that of shear cracks. It is well known that the micro tensile cracks propagate rapidly forming the macro cracks, which corresponds to the number of tensile cracks increasing rapidly, and this process is almost instantaneous. In comparison, the save data interval is greater than that of the increasing rate of the tensile cracks, resulting in the increasing manner of the tensile cracks number is stepwise.

**Figure 11:**
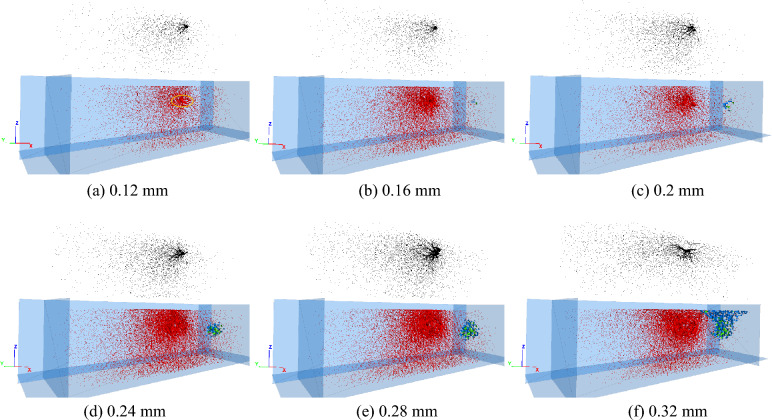
Force chains and microscopic cracks in rock without advanced slotting.

To deeply study the rock-breaking process of the disc cutter, the stroke segment of 0 ~ 0.32 mm was taken for analysis. The internal force chain and crack distribution of the rock under different cutting strokes are shown in Fig. 11, in which the black force chain represents the compressive force chain, the red force chain represents the tensile force chain, the blue cracks represent the tensile cracks, and the green cracks represent the shear cracks. When the cutting stroke of the disc cutter is 0.12 mm, almost no microscopic cracks are initiated, and the rock is in the stage of elastic deformation and has not broken. At this time, the contact area between the rock and the disc cutter (yellow ellipse) produces a staggered compressive force chain and tensile force chain. The force chain spreads around in a dendritic shape and gradually decreases, and the rock at the contact position with the disc cutter bears a larger load. As the disc cutter continues to intrude into the rock, a dense core is gradually formed in the rock area under the action of the cutter, and tensile and shear cracks are initiated and increased, as shown in Fig. 11d,e. Tensile cracks are more significant than shear cracks in the dense core, and the average proportion of tensile cracks is approximately 70%. When the cutting stroke of the disc cutter reaches 0.32 mm, the microscopic cracks in the rock expand to the free surface, the compressive force chain is significantly reduced and weakened, and the cracks from the dense core to the free surface are mainly tensile cracks, as shown in Fig. 11f. This shows that the block fracture of rock under the action of the disc cutter is mainly dominated by tensile failure. In general, during the period from disc cutter cutting rock to the formation of a dense core, the rock breaking of the disc cutter cutting results in tensile and shear failures. As the disc cutter continues to intrude into the rock, the block fracture of the rock is mainly dominated by tensile failure.

### Analysis of the rock breaking mechanism of disc cutter cutting assisted by advanced slotting

**Figure 12:**
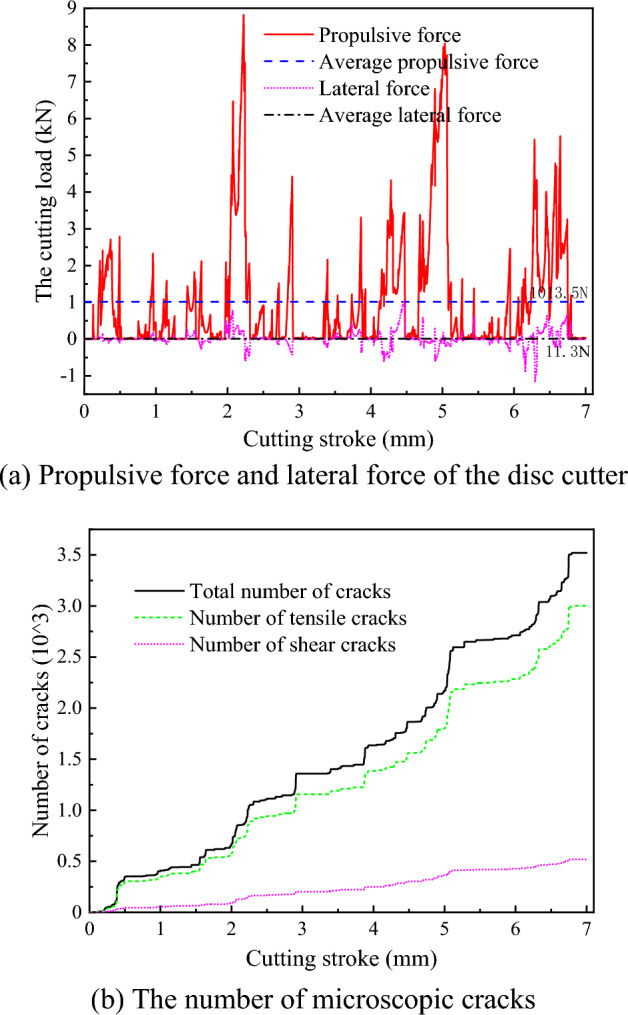
The cutting load and the number of microscopic cracks of the disc cutter cutting rock with advanced slotting.

Keeping the numerical model of the hard rock mass, the rotation speed of the disc cutter and the cutting thickness unchanged, the numerical simulation of rock breaking by disc cutter cutting under an advanced slotting depth of 5 mm was carried out. The load of the disc cutter and the number of microscopic cracks in the rock are shown in Fig. 12. Similarly, the propulsion direction of the disc cutter is always subject to the positive pressure of the rock, the lateral force fluctuates positively and negatively in the x direction, and the average value of the cutter propulsive force is much larger than the average value of the lateral force. The propulsive force of the disc cutter gradually increases to the peak value when the crack is not initiated, and it decreases rapidly when the crack occurs and expands. Compared with the disc cutter cutting rock without advanced slotting, the cutting load and the number of microscopic cracks of the disc cutter cutting rock with advanced slotting are significantly reduced, and the average propulsive force is reduced from 1921.4 to 1013.5 N.

**Figure 13:**
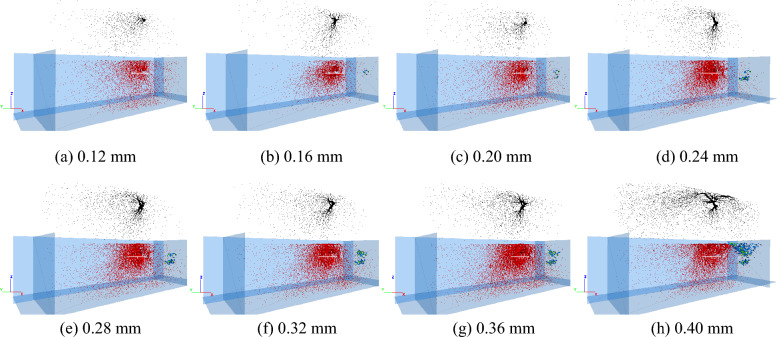
Force chains and microscopic cracks in rock with advanced slotting.

To study the rock-breaking mechanism of disc cutter cutting assisted by advanced slotting, the rock state is analyzed when the disc cutter advancing displacement is 0 ~ 0.4 mm. The internal force chain and crack distribution of the rock when the disc cutter was at different positions are shown in Fig. 13. The meanings of the colors in the figure are the same as those in Fig. 11. When the cutting stroke of the disc cutter is 0.12 mm, an obvious force chain is generated in the rock contact area above the slotting, and the force chain spreads to the free surface in a dendritic shape and gradually decreases, as shown in Fig. 13a. At this time, no microscopic cracks are generated.

In contrast to the rock breaking of disc cutter cutting without advanced slotting, as the disc cutter continues to intrude and cut the rock above and below the slotting, the force chain formed by the contact between the cutter and the rock is V-shaped, as shown in Fig. 13b,c,d,e,f,g. A small dense core, namely, the concentrated zone of compressive force chain, is formed in the contact areas above and below the slotting, and shear and tensile microscopic cracks exist in the area of the dense core. Compared with the unslotted condition, the average proportion of tensile cracks in rock under the condition of advanced slotting increases from 70 to 77%.

There are significant differences in the contact between the disc cutter and the rock: under the condition of no advanced slotting, the contact between the cutter and the rock is similar to the Hertzian contact, and the contact load is mainly concentrated on the cutter edge. Under the condition of advanced slotting, the contact load between the cutter and the rock is mainly concentrated on the wedgy cutter face, the inclined cutter face cuts the rock on the upper side of the slotting, and the pressing force is directed toward the free face of the rock. In addition, according to Figs. 11 and 13, it can be observed that the volume of the dense core of the rock without advanced slotting is significantly larger than that of the rock with advanced slotting, and the reduction of the dense core volume is of great significance to reduce dust production.

## Analysis of influence factors

### Advanced slotting depth

On the basis of the numerical simulations of no advanced slotting and 5 mm of advanced slotting, the numerical simulation of disc cutter cutting rock was carried out under the conditions of 2.5 mm, 7.5 mm, 10 mm and 12.5 mm of advanced slotting. The influence of different advanced slotting depths on the rock breaking performance of the disc cutter was analyzed.

**Figure 14:**
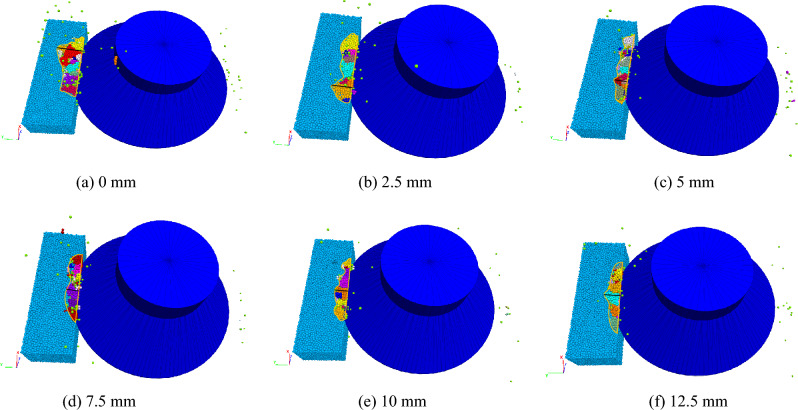
Rock fragmentation under different advanced slotting depths.

The Fig. 14 shows the rock fragmentation at different advanced slotting depths. With the increase in the advanced slotting depth, the width of the rock fragmentation area decreases (the black line in the figure), the acreage of the rock fragmentation area decreases, the fragmentation of the rock fragments decreases, and the volume of broken rock fragments tends to be small and uniform. The main reason for this phenomenon is that when other conditions remain unchanged, the greater the advanced slotting depth is, the greater the stress intensity factor at the tip of the fracture surface under the same load, and the rock is more likely to expand and fracture. The larger the advanced slotting depth is, the lower the bearing capacity and bending resistance of the rock above the fracture surface, which makes the upper rock easy to bend and fracture, which is also the reason for the reduction in rock lumpiness under a large slotting depth.

**Figure 15:**
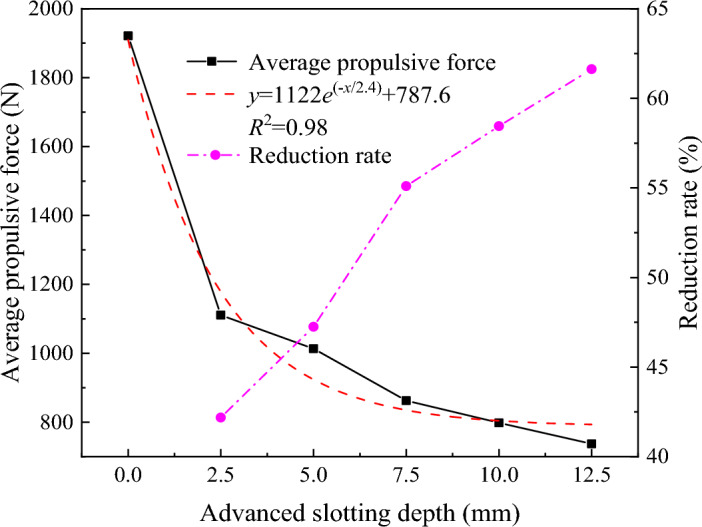
The relationship between the average propulsive force and descent rate and advanced slotting depth.

To intuitively analyze the influence of the advanced slotting depth on the rock breaking performance of the disc cutter, considering that the lateral force of the disc cutter is much smaller than the propulsive force, taking the average propulsive force of the cutter as the load evaluation index, the variation in the average propulsive force of the cutter with the advanced slotting depth is shown in Fig. 15. The average propulsive force of the disc cutter decreases exponentially with increasing advanced slotting depth, the correlation coefficient is greater than 0.98, and the exponential fitting error is small. When the advanced slotting depth is greater than 5 mm, the average propulsive force of the disc cutter tends to remain unchanged, indicating that there is a critical value of the advanced slotting depth. When the slotting depth exceeds this value, continuing to increase the slotting depth has no significant effect on reducing the average propulsive force of the cutter. The average propulsive force of the disc cutter without advanced slotting is approximately 1921.4 N, and the average propulsive force of the disc cutter with slotting depths of 2.5 mm and 12.5 mm is 1110.9 N and 737.3 N, respectively, it can be seen that advanced slotting can significantly reduce the rock-breaking load of the disc cutter, reduce the requirements for the structural strength and wear resistance of the disc cutter, and provide a feasible method for the mechanized continuous cutting of superhard rock. The advanced slotting methods currently include saw cutting and water jets. Advanced slotting of approximately 0–10 mm can be realized by integrating the water jet into the disc cutter^28–31^.

However, it is not comprehensive to evaluate the rock breaking performance of the disc cutter only by the average propulsive force. To further study the influence of the advanced slotting depth on the rock breaking performance of the disc cutter, the rock-breaking specific energy consumption was introduced as a performance evaluation index^32,33^. The rock-breaking specific energy consumption refers to the energy consumed by the disc cutter to break the unit volume of rock. The smaller the specific energy consumption is, the better the comprehensive rock breaking performance of the disc cutter. In this study, the energy consumed by the disc cutter for rock breaking mainly includes the feeding work and the rotating work of the cutter. The specific energy consumption for rock breaking is expressed as follows:

8 $$E_{{\text{s}}} = \frac{{W_{{\text{l}}} + W_{{\text{r}}} }}{V} = \frac{{\mathop \smallint \nolimits_{0}^{{L_{s} }} F{\text{d}}l + \mathop \smallint \nolimits_{0}^{\emptyset } T{\text{d}}\theta }}{V}$$

where ${E}_{s}$ is the rock breaking specific energy consumption, MJ/m^3^; ${W}_{\mathrm{l}}$ is the feeding work of the disc cutter, J; ${W}_{\mathrm{r}}$ is the rotating work of the disc cutter, J; $V$ is the rock breaking volume, m^3^; $F$ is the propulsive force of the disc cutter, N; $T$ is the torque of the disc cutter, N m; ${L}_{s}$ is the critical feed stroke of the disc cutter, m; $l$ is the displacement of the disc cutter, m; $\varnothing$ is the maximum rotation radian of the disc cutter, rad; and $\theta$ is the rotation radian of the disc cutter, rad.

**Figure 16:**
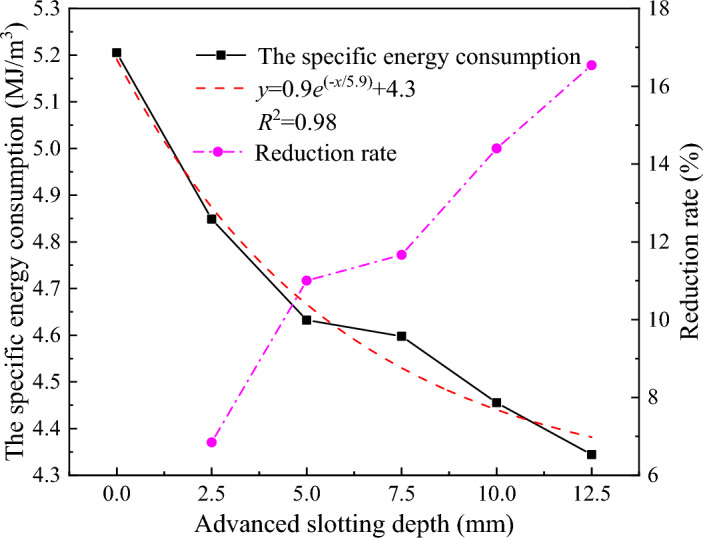
Variation curve of the specific energy consumption of rock breaking with advanced slotting depth.

The relationship between the rock breaking specific energy consumption and the advanced slotting depth is shown in Fig. 16. It can be seen that the increase in advanced slotting depth leads the rock breaking specific energy consumption of the disc cutter to decrease exponentially. The specific energy consumption of the disc cutter without the advanced slotting is 5.2 MJ/m^3^, when the advanced slotting depth is 2.5 mm and 12.5 mm, the specific energy consumption of the disc cutter is 4.8 MJ/m^3^ and 4.3 MJ/m^3^, respectively, indicating that the auxiliary effect of advanced slotting can effectively reduce the rock breaking specific energy consumption of the disc cutter.

The fracture surface formed by the advanced slotting can have a weakening effect on the surrounding rock mass, reduce the rock strength and the dust production, make the rock easier to break, reduce the rock breaking load of the disc cutter, and thus improve the rock breaking performance of the disc cutter. Considering the average propulsive force of the disc cutter and the specific energy consumption of rock breaking, it can be seen that when the advanced slotting depth exceeds 5 mm, continuing to increase the advanced slotting depth has relatively little improvement in the rock breaking performance of the disc cutter. In addition, the greater the depth of actual water jet cutting rock is, the higher the requirement of jet pressure, the greater the difficulty, and the higher the specific energy consumption. Therefore, the optimal depth of disc cutter cutting rock assisted by advanced slotting is 5–7.5 mm.

### Cutting thickness

Under the condition that the feed speed of the disc cutter is 0.1 m/s, the rotation speed is 50 rad/s, and the advanced slotting depth is 7.5 mm, the cutting thicknesses are selected as 7.5 mm, 10 mm, 12.5 mm, 15 mm, and 17.5 mm, respectively, and the effect of different cutting thicknesses on the rock breaking performance of the disc cutter was studied.

**Figure 17:**
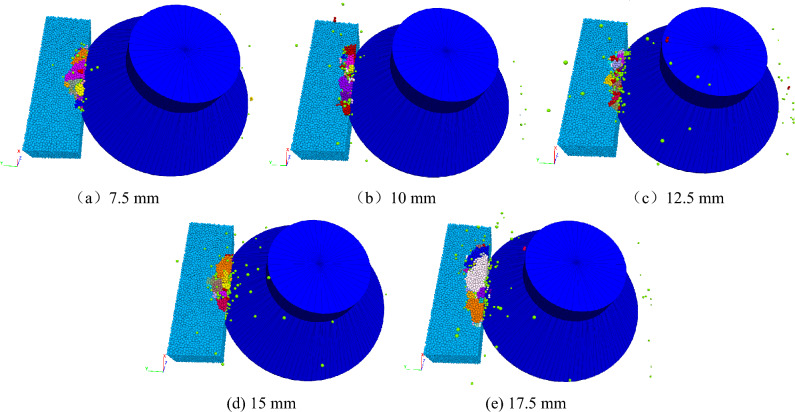
Rock fragmentation under different cutting thicknesses.

The Fig. 17 shows the rock fracture under different cutting thicknesses. In general, the rock fracture volume and lumpiness increase with increasing cutting thickness of the disc cutter. The reason is that the greater the cutting thickness is, the more bonding bonds between the particles above the slotting, the stronger the bonding effect, the better the rock integrity above the slotting, and the stronger the anti-fracture ability, so rock can withstand greater force, and the cracks can be initiated from the tip of the advanced slotting, eventually forming large fragments.

**Figure 18:**
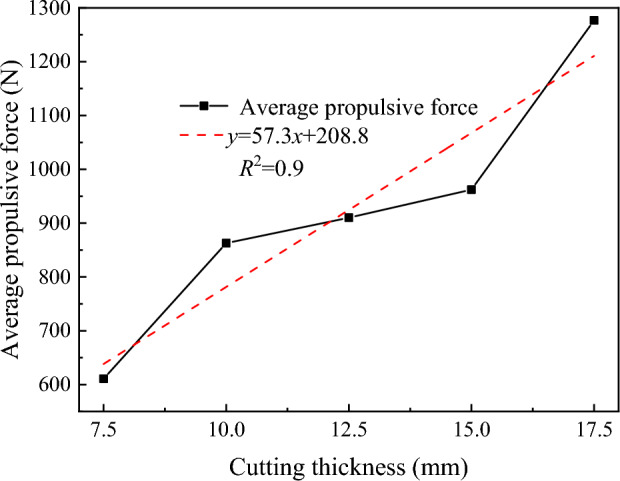
The relationship between the average propulsive force and cutting thickness.

The relationship between the average propulsive force of the disc cutter and the cutting thickness is shown in Fig. 18. It can be seen that the average propulsive force of the disc cutter increases with as the cutting thickness increases, and there is a good linear relationship (R^2^ = 0.94), which indirectly verifies that the greater the cutting thickness mentioned above is, the better the integrity of the rock above the kerf, the stronger the anti-fracture ability, and the greater the propulsive force of the disc cutter required to break the rock above the slotting.

**Figure 19:**
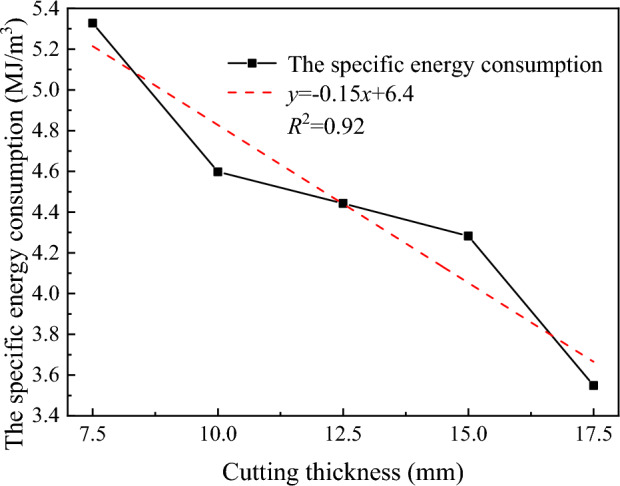
Variation curve of the specific energy consumption of rock breaking with cutting thickness.

The relationship between the rock breaking specific energy consumption and the cutting thickness is shown in Fig. 19. The rock breaking specific energy consumption decreases with as the cutting thickness increases, and there is a good linear relationship (R^2^ = 0.92). When the cutting thickness is 17.5 mm, the rock breaking specific energy consumption is 33.4% lower than that when the cutting thickness is 7.5 mm, indicating that the larger the rock cutting thickness is, the lower the specific energy consumption of the disc cutter is. It has been confirmed above that advanced slotting can significantly reduce the load of the disc cutter. Within the range of the structural strength of the disc cutter, the auxiliary effect of advanced slotting provides an effective method for increasing the cutting thickness and reducing the energy consumption of the cutter.

In general, increasing the cutting thickness has advantages and disadvantages. On the one hand, the larger the cutting thickness is, the greater the load of the disc cutter in rock breaking, and the worse the stress condition. On the other hand, increasing the cutting thickness can increase the broken volume of rock, reduce the rock breaking specific energy consumption, and improve the rock lumpiness. Therefore, within the scope of the structural strength of the disc cutter, the rock breaking operation of the disc cutter should be carried out with a large cutting thickness as much as possible to give full play to the auxiliary function of the advanced slotting and the structural strength of the disc cutter.

### Rock strength

On the basis of the numerical simulation of disc cutter cutting hard rock (bluestone, compressive strength 122 MPa), the cutting thickness is set to 10 mm and the advanced slotting depth is 5 mm, the numerical simulation of disc cutter cutting three kinds of rocks (20 MPa concrete, 56.5 MPa sandstone, 82 MPa granite) is carried out to study the effect of different rock strengths on the rock breaking performance of disc cutter cutting.

**Figure 20:**
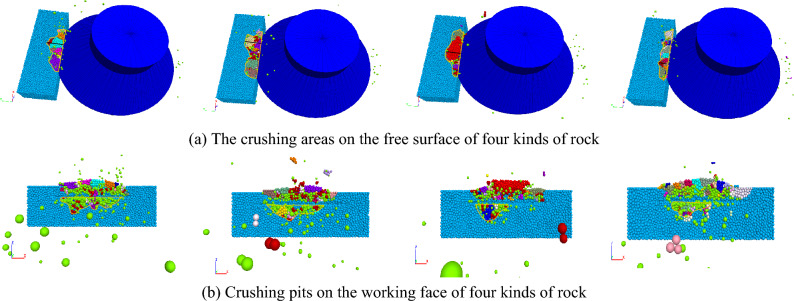
The breaking conditions of rocks with different strengths.

The Fig. 20 shows the breaking condition of rocks with different strengths under advanced slotting. Figure 20a shows the crushing areas on the free surface of concrete, sandstone, granite and bluestone in turn, and Fig. 20b shows their corresponding crushing pits on the working face. Under the same parameter conditions, with increasing rock strength, the width of rock fragments on the free surface decreases, and the area of caving pits decreases, indicating that the crushed volume of concrete and sandstone is higher than that of granite and bluestone. The lower the rock strength is, the lower its compressive and tensile capacity and the crack initiation strength of the advanced slotting tip, and the easier the rock is to break.

**Figure 21:**
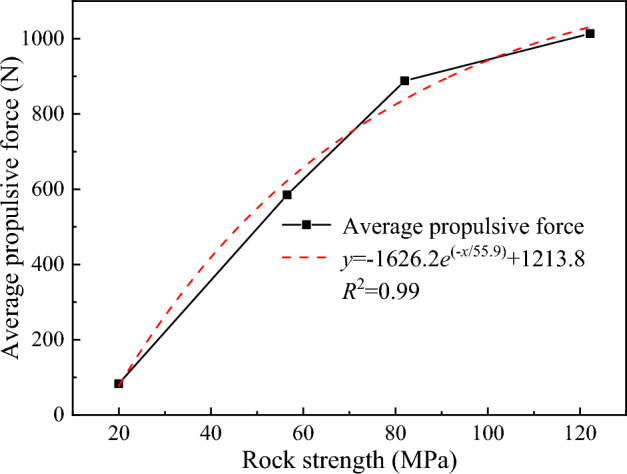
The relationship between the average propulsive force and rock strength.

**Figure 22:**
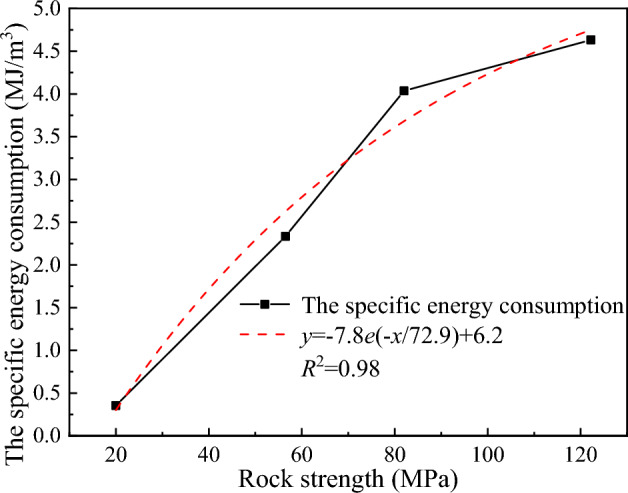
Variation curve of the specific energy consumption of rock breaking with rock strength.

The variation in the average propulsive force of the disc cutter under different rock strength conditions is shown in Fig. 21. As the rock strength increases, the average propulsive force required by the disc cutter to break the rock increases, but the growth rate gradually decreases. The reason is that as the rock strength increases, the disc cutter has more difficulty penetrating the rock, and it takes a larger load to break the hard rock. Figure 22 shows the change in specific energy consumption of the disc cutter for breaking rocks with different strengths. It can be seen that the greater the rock strength is, the higher the specific energy consumption of rock breaking, while the growth rate of specific energy consumption gradually decreases.

Overall, the change law of cutting load and rock breaking specific energy consumption for cutting rocks with different strengths is in line with reality. When the rock strength is greater than 82 MPa, the average load and specific energy consumption growth rate are further reduced, indicating that the load and specific energy consumption for crushing granite and bluestone tend to be similar. Therefore, within the allowable range of the structural strength of the disc cutter, the rock breaking of the disc cutter assisted by advanced slotting is more suitable for cutting rock with high strength.

### Rotation speed

Under the condition that the feed speed of the disc cutter is 0.1 m/s, the cutting thickness is 10 mm, the advanced slotting depth is 5 mm, the cutting object is bluestone, and the rotation speeds of the disc cutter are selected as 25 rad/s, 50 rad/s, 75 rad/s, 100 rad/s, 150 rad/s and 175 rad/s, the effect of different rotation speeds on the rock breaking performance of the disc cutter was studied.

**Figure 23:**
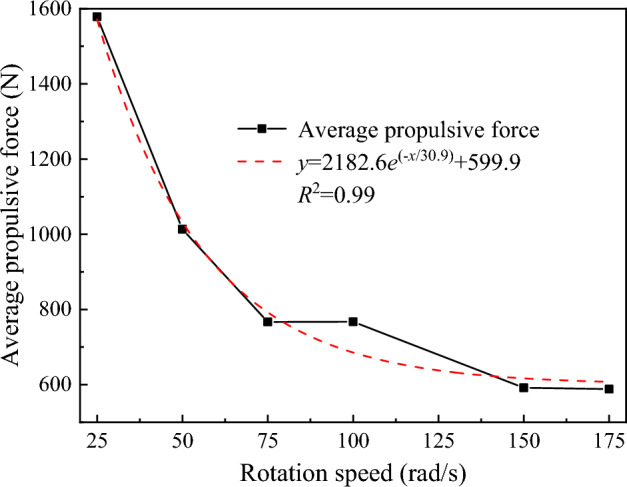
The relationship between the average propulsive force and rotation speed.

The Fig. 23 shows the relationship between the average propulsive force of the disc cutter and the rotation speed. As the rotation speed increases, the average propulsive force of the disc cutter decreases exponentially as a whole, and the decline rate gradually approaches zero. The reason is that the higher the rotation speed of the disc cutter is, the stronger the rotational effect of the disc cutter in the process of cutting rock, and the greater the speed applied by the disc cutter to the contact particles in the dense core area, which leads the failed particles to fly away at a higher speed instead of stagnating in front of the cutter and hinder rock breaking. The number of particles in the dense core area is reduced, so the stress transmitted to the surrounding rock is reduced, the initiation and propagation of cracks are reduced, and the stress of the disc cutter is improved.

**Figure 24:**
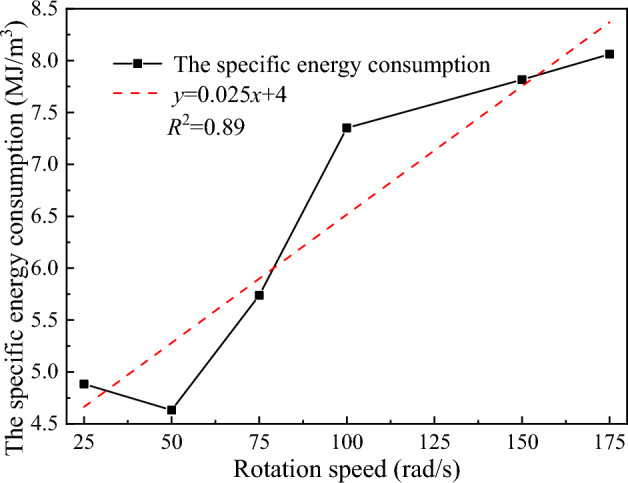
Variation curve of the specific energy consumption of rock breaking with rotation speed.

The variation curve of the specific energy consumption of rock breaking against the rotation speed is shown in Fig. 24. It can be seen that the rock breaking specific energy consumption of the disc cutter increases as the overall rotation speed increases, showing a good exponential relationship. From the comprehensive consideration of the average propulsive force and the rock breaking specific energy consumption, when the rotating speed of the disc cutter takes a larger value, the rock breaking load of the disc cutter is small, but the corresponding rock breaking specific energy consumption is large, and the high speed has higher requirements on the driving equipment. Therefore, within the allowable range of the structural strength of the disc cutter, the rock breaking operation should be carried out at a low speed as far as possible to give full play to the performance of the disc cutter and reduce the energy consumption of rock breaking.

## Conclusion

A) Based on the parallel bond constitutive model, the microparameters of the rock discrete element numerical model were obtained by "trial and error method", and the three-dimensional discrete element numerical model of rock breaking by the disc cutter was established. The load and rock fragment size of the cutting test and numerical simulation are compared and analyzed, which confirms the correctness of the established numerical model and provides a reliable method for research on rock breaking of disc cutter assisted by advanced slotting.
B) When there is no advanced slotting, the contact between the disc cutter and the rock is similar to the Hertzian contact, the contact force chain is dendritic, and the contact load is mainly concentrated on the cutter edge. When there is advanced slotting, the contact load between the cutter and the rock is mainly concentrated on the wedgy cutter face, the inclined cutter face cuts the rock on the upper side of the slotting, the pressing force is directed toward the free face of the rock, and the strength requirement for the cutter is reduced.
C) The average propulsive force of the disc cutter decreases exponentially with the increase of advanced slotting depth; the greater the advanced slotting depth is, the lower the bearing capacity and bending resistance of the rock above the fracture surface. There is a critical value for the advanced slotting depth; when the slotting depth exceeds this value, continuing to increase the slotting depth has no significant effect on reducing the average propulsive force of the disc cutter. When the advanced slotting depth is 12.5 mm, the average propulsive force and specific energy consumption of the disc cutter are reduced by 61.6% and 16.5%, respectively.
D) The average propulsive force of the disc cutter and the rock breaking specific energy consumption increase as the rock strength increases. When the rock strength is greater than 82 MPa, the load and the specific energy consumption for crushing granite and bluestone tend to be similar. Therefore, within the allowable range of the structural strength of the disc cutter, the rock breaking of the disc cutter assisted by advanced slotting is more suitable for cutting rock with high strength. As the rotation speed of the disc cutter increases, the average propulsive force and the specific energy consumption of the disc cutter decrease and increase exponentially, respectively. The average propulsive force and the specific energy consumption of the disc cutter increase and decrease linearly with the cutting thickness, respectively.

## Data Availability

The datasets used and/or analysed during the current study available from the corresponding author on reasonable request.
